# Supplementary material for: Citizen science provides a reliable and scalable tool to track disease-carrying mosquitoes
Source: Nat Commun. 2017 Oct 24;8:916. doi: 10.1038/s41467-017-00914-9 (PMC5655677; doi:10.1038/s41467-017-00914-9)
Supplement: Supplementary file 1 — Supplementary Information [file 41467_2017_914_MOESM1_ESM.pdf]

Supplementary Table 1. Comparison of reporting propensity models, in which the outcome variable is presence or absence of a report sent from a given participant a given day. For main effects, values in parentheses below each estimate are standard errors, calculated as mean absolute deviation of the posterior distribution. All models include random intercepts for participant, capturing intrinsic participant motivation. ELPD is Expected Log Pointwise Predictive Density, estimated with leave-one out cross validation (LOO), with standard errors in parentheses calculated as standard deviation of the components that are summed to form the ELPD. N is the number of observations (participant-days) used in each model, which is 10,000 in all cases, as this was the population size set in the resampling described in the Methods Section.

	M0	M1	M2	M3
participation time (days)		-0.01	-0.01	0.004
		(0.002)	(0.001)	(0.004)
participation time (days) sq.			0.02	0.01
			(0.003)	(0.003)
participation time (days) cu				-0.03
				(0.01)
ELPD	-509	-486	-469	-464
	(44)	(42)	(41)	(41)
N	10,000	10,000	10,000	10,000

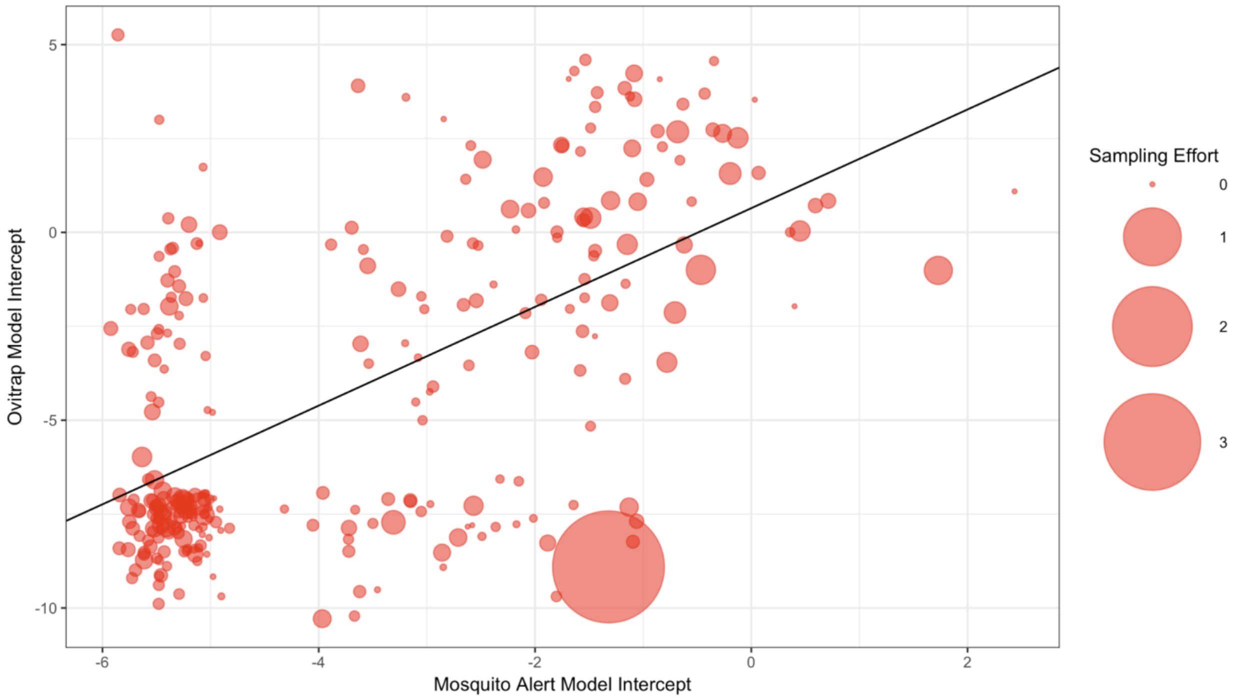

Supplementary Figure 1. Comparison of Mosquito Alert and ovitrap estimates of random intercepts for overlapping prediction cells during 2014-2015, using models O5 and MA5 (Tab. 2). The random intercept for each cell represents that cell's intrinsic tiger mosquito risk (human-mosquito encounter probability in the case of the Mosquito Alert model; egg probability in the case of the ovitrap model) unconditional on sampling effort or seasonality. Circles show random intercepts from each model, with size proportional to Mosquito Alert sampling effort and line indicating OLS regression (slope=1.31, R-sq.=0.36).

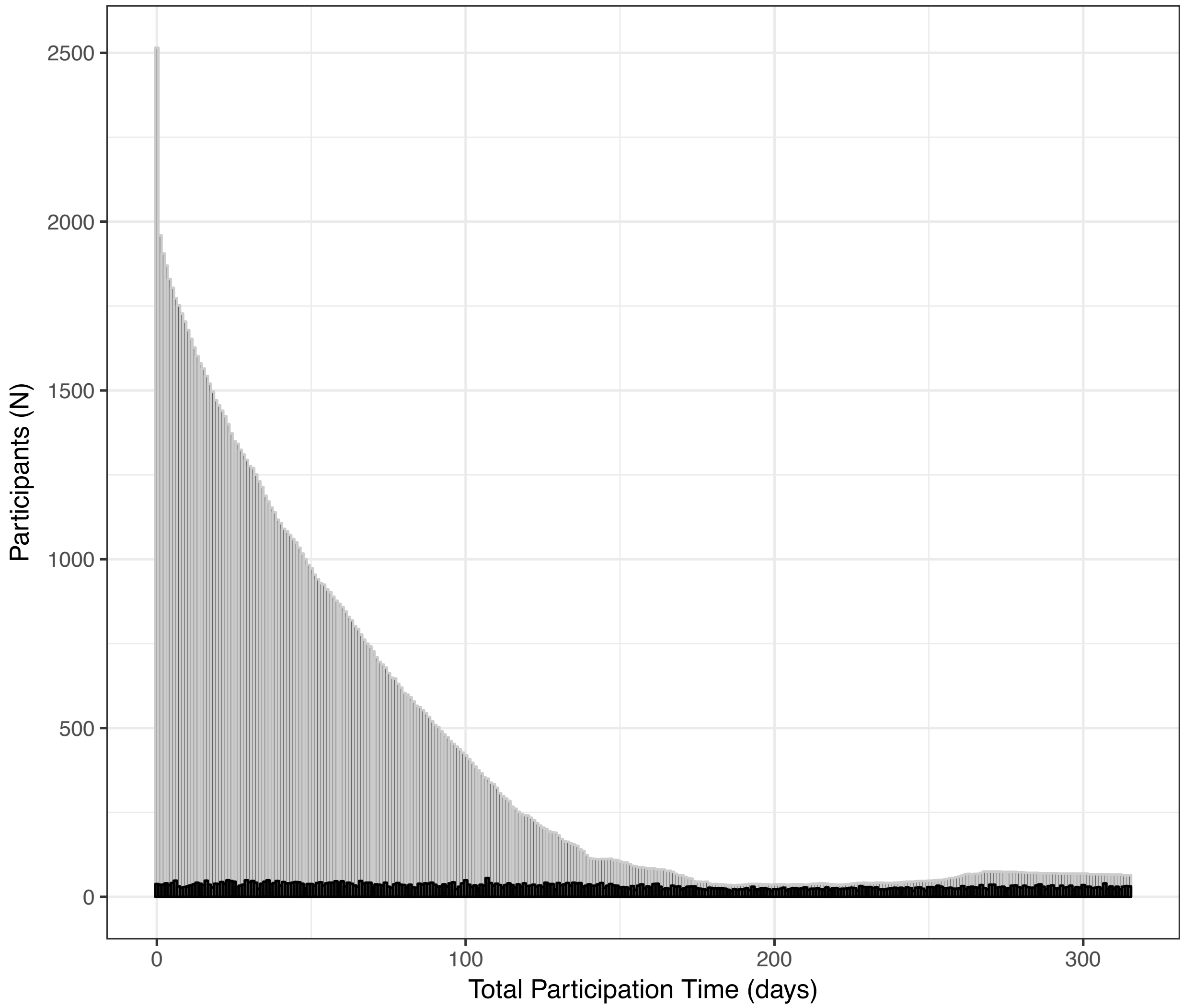

Supplementary Figure 2. Resampling of data for reporting propensity model. Grey bars show distribution of participants' total participation times in original data; black bars show distribution after resampling to improve balance. The resampling, to reduce covariate imbalance in the data, was done by dropping the highest 5% and lowest 5% of the participation times and then resampling the data with sampling weights inverse to the proportion of each total participation time (in days) in the remaining data. We then used the resampled data to get better estimates of the reporting propensity of participants.

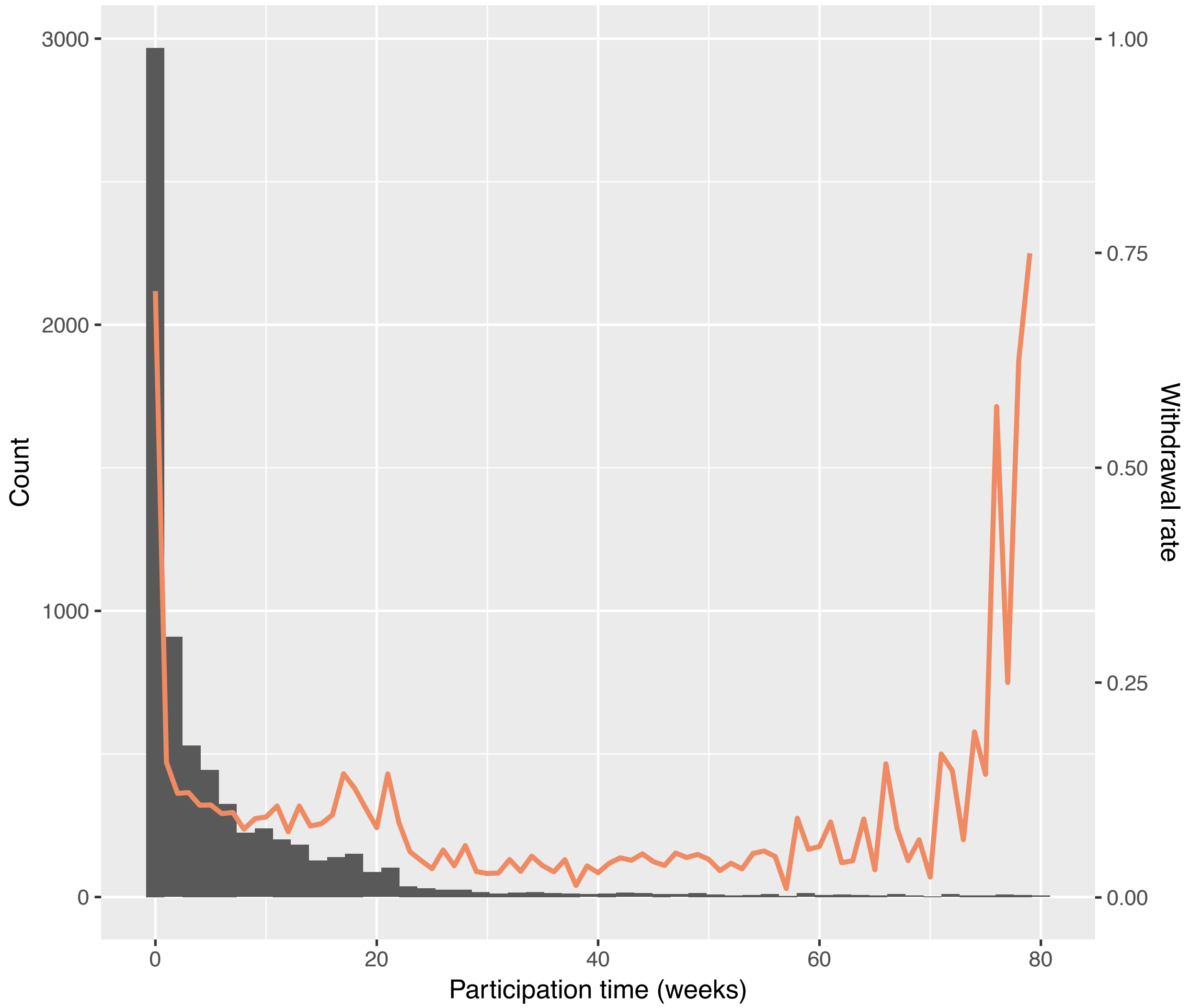

Supplementary Figure 3. Participation times and withdrawal rates. Grey bars show number of participants (left axis) by total participation time (using a histogram with 50 bins). Brown line shows participation-time-specific withdrawal rates (right axis), meaning withdrawal rates conditional on reaching a given participation time (analogous to age-specific mortality rates), calculated on a weekly basis.
