# Supplementary material for: Citizen science provides a reliable and scalable tool to track disease-carrying mosquitoes
Source: Nat Commun. 2017 Oct 24;8:916. doi: 10.1038/s41467-017-00914-9 (PMC5655677; doi:10.1038/s41467-017-00914-9)
Supplement: Supplementary file 3 — Description of Additional Supplementary Files [file 41467_2017_914_MOESM3_ESM.pdf]

Description of Supplementary Files

File name: Supplementary Movie 1
Description: Animated daily municipal alert probabilities in Spain, based on 2014-15 Mosquito Alert reliable reports. Grey municipalities not sampled by Mosquito Alert participants during this period. Each frame of the animation shows estimated alert probabilities for a given day between April and November, calculated for the two-week period preceding that day.

File name: Supplementary Movie 2
Description: Animated daily municipal alert probabilities in Catalonia, based on 2014-15 Mosquito Alert reliable reports. Grey municipalities not sampled by Mosquito Alert participants during this period. Each frame of the animation shows estimated alert probabilities for a given day between April and November, calculated for the two-week period preceding that day.
