# Supplementary figures and images for: Citizen science provides a reliable and scalable tool to track disease-carrying mosquitoes
Source: Nat Commun. 2017 Oct 24;8:916. doi: 10.1038/s41467-017-00914-9 (PMC5655677; doi:10.1038/s41467-017-00914-9)

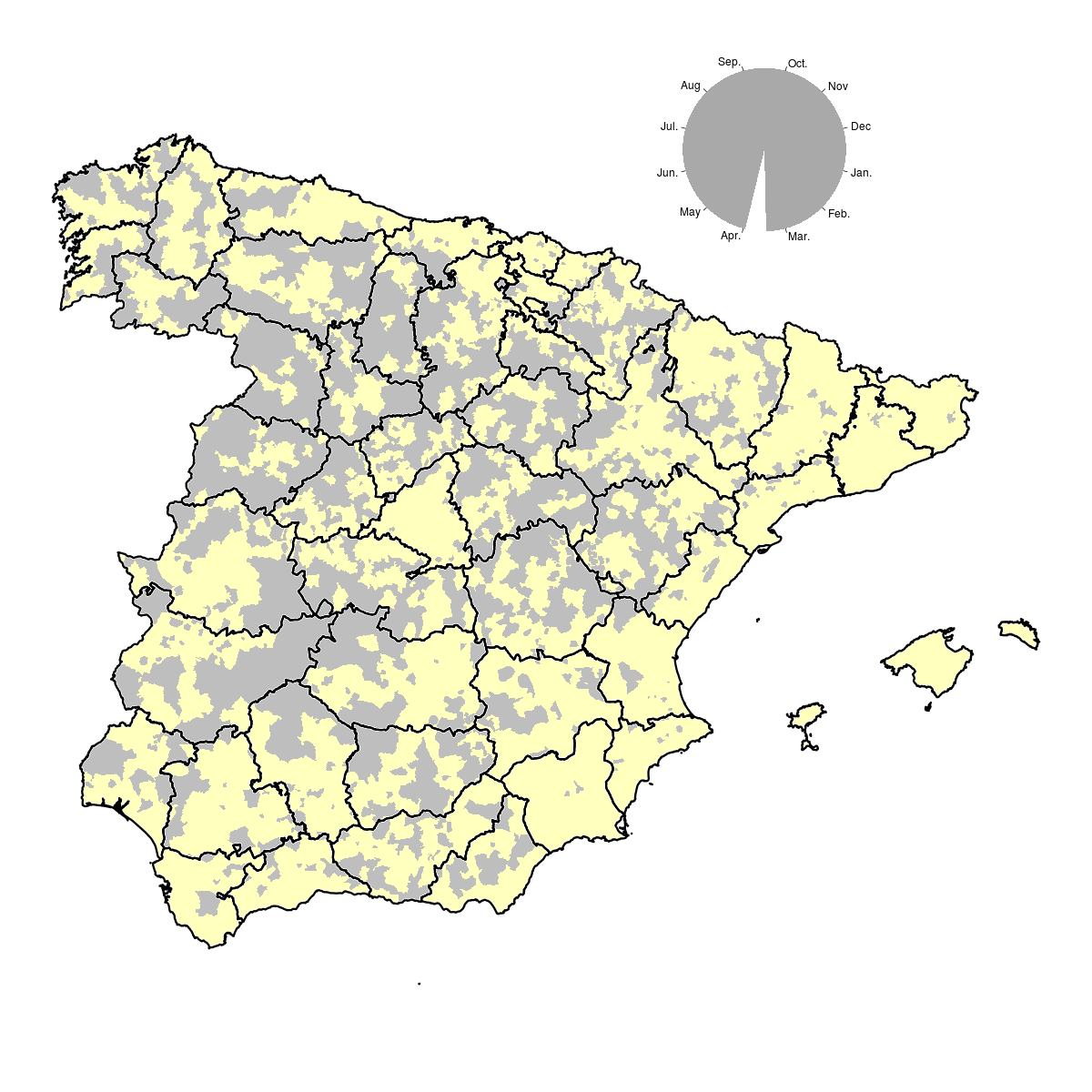

Supplement: Supplementary file 4 — Supplementary Movie 1 [file 41467_2017_914_MOESM4_ESM.gif]

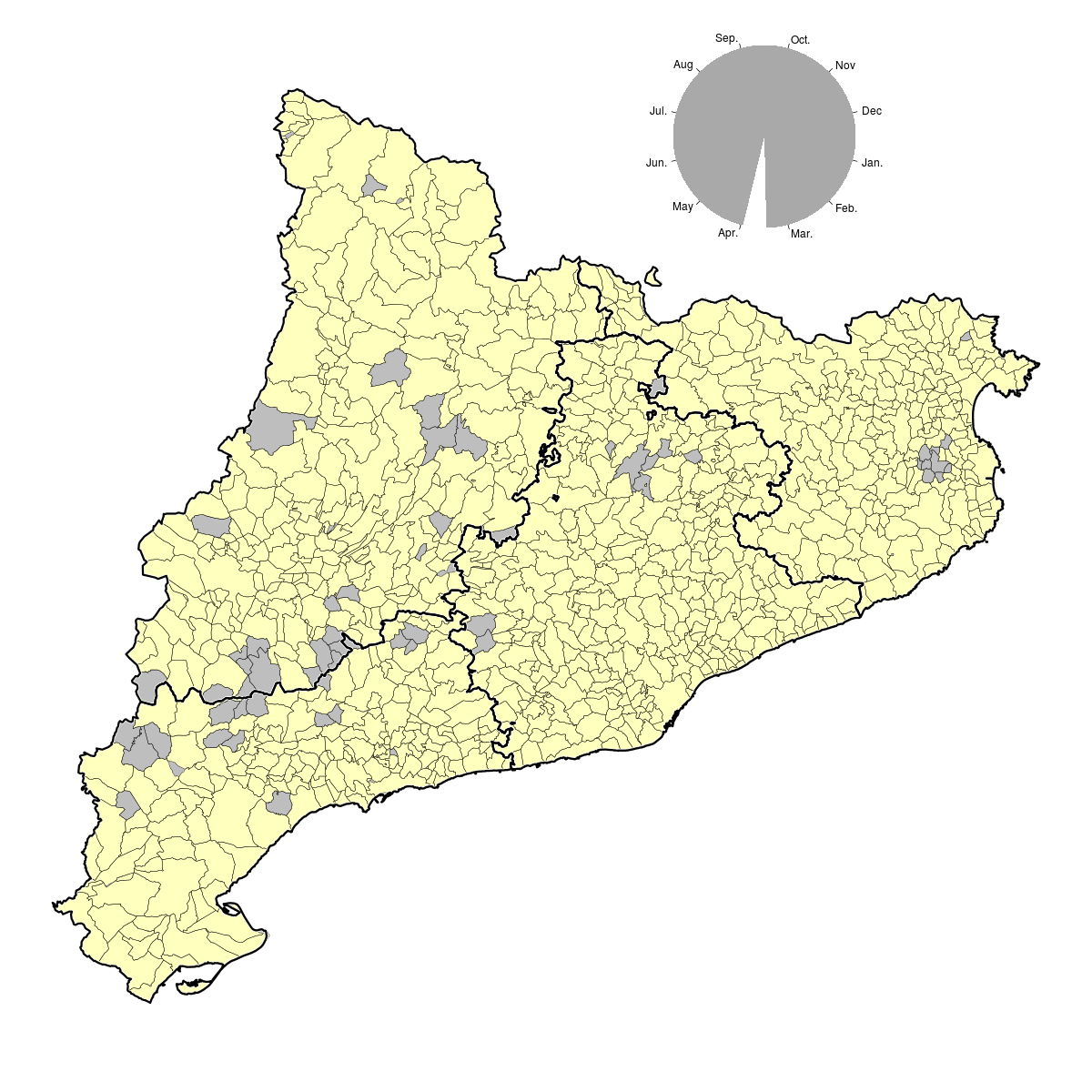

Supplement: Supplementary file 5 — Supplementary Movie 2 [file 41467_2017_914_MOESM5_ESM.gif]
